# Water‐Mediated Epoxy/Surface Adhesion: Understanding the Interphase Region

**DOI:** 10.1002/chem.202202483

**Published:** 2022-10-01

**Authors:** Charlie R. Wand, Simon Gibbon, Peter Visser, Flor R. Siperstein

**Affiliations:** ^1^ Natural Sciences College of Engineering Mathematics and Physical Science Streatham Campus University of Exeter North Park Road EX4 4QF Exeter UK; ^2^ Department of Chemical Engineering The University of Manchester Oxford Road M13 9PL Manchester UK; ^3^ Corrosion and Protection Centre School of Materials The University of Manchester Oxford Road M13 9PL Manchester UK; ^4^ AkzoNobel Expertise Center Corrosion Rijksstraatweg 31 2171 AJ Sassenheim The Netherlands

**Keywords:** epoxy resin, interfaces, molecular dynamics, polymers, simulation

## Abstract

Epoxy resins coatings are commonly found in corrosion protection coatings but the presence of water can affect their adhesion to the substrate, often weakening the adhesion of the coating to the solid, reducing its efficiency. Nevertheless, small amounts of water can enhance the epoxy/substrate interactions. In this work, the interphase region of an epoxy precursor and metal oxide substrates is investigated using molecular simulations and it is found that water accumulates between the epoxy layer and the solid substrate. At high water concentrations (9 wt %) the interaction between the epoxy precursor and the solid surface is weakened regardless of the nature of the solid, but at low water concentrations the nature of the solid surface becomes important. For hematite, the presence of water decreases the strength of adhesion but for goethite the presence of a small amount of water (3 wt %) enhances the adhesion to the surface resulting in a densification at the interface.

## Graphical abstract

**Epoxy resin coatings** are commonly used to protect metal surfaces from corrosion. Molecular simulations are used to investigate the adhesion in an epoxy resin precursor to hematite and goethite surfaces, as a proxy for steel surfaces and the effect of water ingress. For hematite water always decreases the adhesion whilst for goethite a small amount of water increases adhesion to the surface.

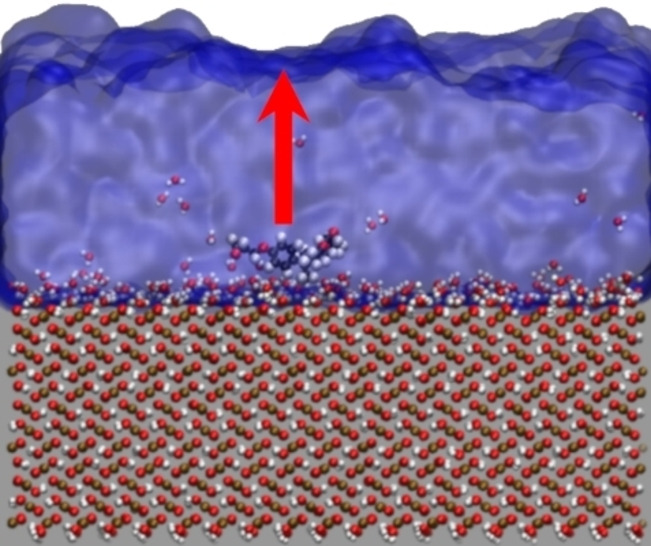

## Introduction

Epoxy resins are used as both adhesives and protective coatings with applications across a range of industries including automotive, aerospace, freight, and the oil/gas industry.[^1^, ^2^] The ubiquitous use of epoxy resins arises from their good heat and chemical resistance, favorable mechanical properties, and good adhesion to a range of substrates. However, failure of the epoxy resin can result in joint failure and/or exposure of the underlying substrate to the environment making it vulnerable to corrosion, shortening the lifespan of the substrate. The cost of corrosion worldwide has been estimated to be US$2.5trillion.^[3]^ Electrochemical corrosion is the electrochemical oxidation of a metal and requires three things to be present; an electron acceptor, an exposed metal surface, and an electrolyte such as water. Therefore, by removing one of these factors such as the water, corrosion can be prevented. Unfortunately, it is not possible to completely stop moisture ingress and moisture has been found to decrease the adhesion,[^4^, ^5^, ^6^] cause yellowing^[7]^ and cracking.^[8]^ The adhesion mechanism at a molecular‐level is still not well understood and can provide important insights into the formulation of better coatings and adhesives. Recently spectroscopic methods have been employed to look at the buried interface including photothermal infrared (PTIR), electron energy loss spectroscopy (EELS), electrochemical impedance spectroscopy (EIS) and atomic force microscopy with infrared (AFM‐IR)[^9^, ^10^, ^11^] and have found that corrosion can initiate at the buried interface. Morsch et al.^[9]^ have found that the oxidation at the buried interface is consistent with diffusion limited oxidation that is controlled by oxygen transport along the polymer‐metal interface.

Computational approaches have been used to complement experimental methods and can be used to assess the strength of interaction between an epoxy precursor and the solid substrate. Quantum calculations can accurately model the charge transfer in chemisorption and have been widely used to study alumina[^12^, ^13^] and other surfaces such as cyano‐modified gold,^[14]^ iron,^[15]^ silica^[16]^ and iron oxides.[^17^, ^18^, ^19^, ^20^, ^21^, ^22^] However, due to the computational resources required these calculations are limited to a small number of atoms. Ogata and Takahashi have addressed this by the use of a hybrid quantum‐classical simulation method to extend the size of the system and investigate the influence of water on the adhesion between alumina and an epoxy resin and find that the water mediates bond formation between the epoxy and the substrate.^[23]^ Classical simulation approaches such as molecular dynamics (MD) can be used to study larger systems by increasing the system's length and time scales. Here we consider hematite (α‐Fe_2_O_3_) (0001) surface and goethite (α‐FeOOH) (100) surface as a proxy for the oxide formation on iron and steel.[^24^, ^25^, ^26^] Previous quantum studies have found that epoxy amine coatings on iron and iron oxide/oxyhydroxide surfaces do not chemisorb making them an ideal system for MD simulations.^[15]^

**Figure 1:**
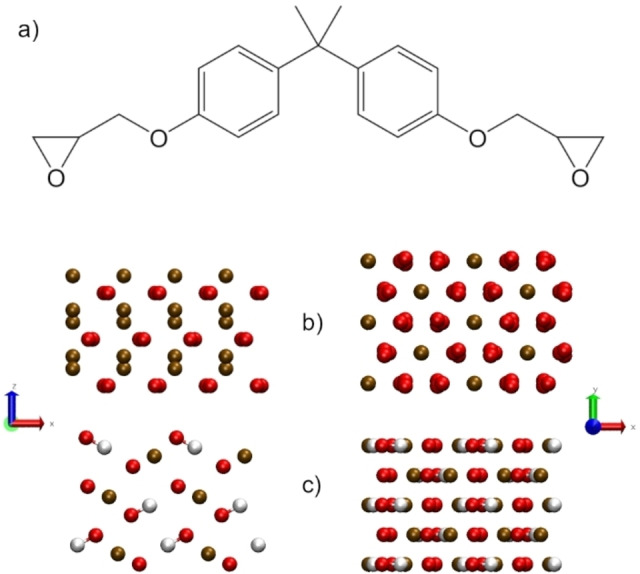
a) Schematic of the epoxy coating precursor, DGEBA. The crystalline structures of b) hematite and c) goethite where oxygen is shown in red, iron in brown and hydrogen in white.

We have previously investigated the binding strength of single molecules of common epoxy‐amine resin components including Bisphenol A diglycidyl ether (DGEBA) (Figure 1a) on iron oxide and oxyhydroxide surfaces[^21^, ^22^] and found that the binding on hematite and magnetite (Fe_3_O_4_) is primarily driven by electrostatic interactions between the electronegative atoms in the epoxy matrix and the positive iron sites in the surface. However, in the case of goethite the electronegative atoms in the epoxy matrix can form additional hydrogen bonds with the surface hydroxyl groups. Whilst these results elucidate the binding between the different surfaces and the epoxy precursor, they do not take into account *intra*‐epoxy interactions that are important when considering the influence of moisture on the binding. In this paper we focus on the effect of water on the binding of a DGEBA molecule within a film on hematite (Figure 1b) and goethite (Figure 1c).

We constructed a layer of DGEBA on the selected surface containing 360 molecules, and different water contents (0, 3, and 9 wt % water), allowing the system to equilibrate at 350 K (unconstrained simulations). We use the OPLS‐AA force field^[27]^ for DGEBA with SPC water.^[28]^ To model the solid slab we use the CLAYFF force field, with a modification by Kerisit.[^29^, ^30^] More details of the simulation set‐up including the charges can be found in the Supporting Information. Note that we start from a system with the water at the interface and allow it to fully equilibrate before analyzing the system. From these we then calculate the potential of mean force (PMF) for removing a single DGEBA molecule from the surface at 300 K. The system properties for the unconstrained and PMF systems are very similar, and a comparison can be found in the Supporting Information.

## Results and Discussion

**Figure 2:**
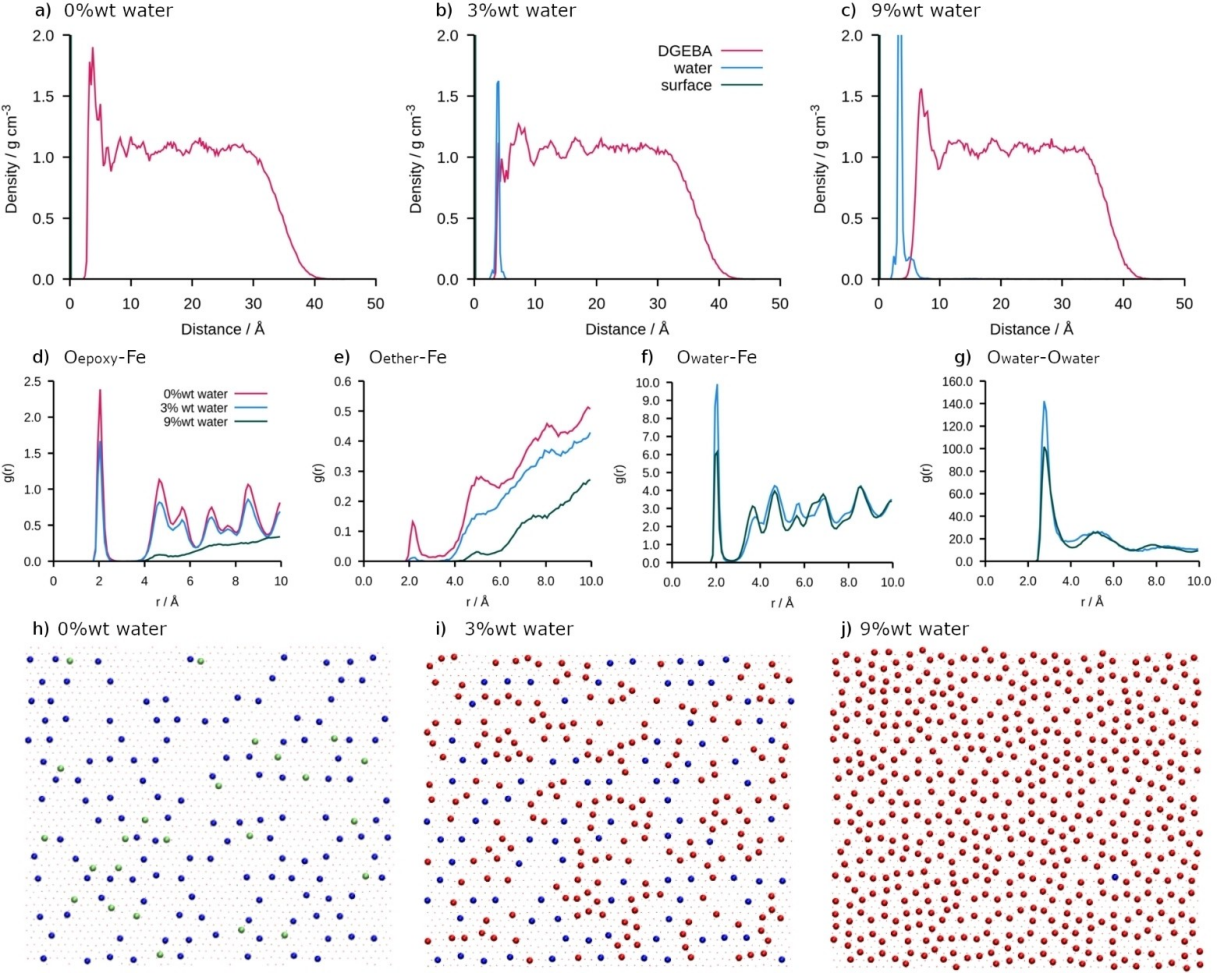
Characterisation of the hematite/DGEBA interface including density profiles, radial distribution functions between selected pairs of atoms, and the snapshots of the position of interfacial oxygen atoms. Density in *z*‐direction for a) 0 wt %, b) 3 wt % and c) 9 wt % water. Radial distribution functions for d) O_epoxy_−Fe e) O_ether_−Fe, f) O_water_−Fe, and g) O_water_−O_water_. Snapshots of the surface oxygen for h) 0 wt %, i) 3 wt %, and j) 9 wt %. The interfacial oxygen are defined as oxygen atoms within 4 Å of the surface. Epoxide oxygen in DGEBA are shown in blue, ether oxygen in DGEBA in green and water oxygen in red.

The dry film of DGEBA on hematite (0 wt % water) has an initial peak in density profile along *z* at approximately 3 Å due to favorable interactions between the DGEBA and the positive iron sites in the surface. After this initial peak the density fluctuates around the bulk density of 1.07 g cm^−3^. Preliminary simulations have validated the model against literature data and details can be found in the Supporting Information. At both 3 wt % and 9 wt % water, the water is located at the interface between the solid and the epoxy precursor, in agreement with experimental studies that suggest that the water is located between the epoxy and the surface[^29^, ^30^] and there is little penetration of the water into the epoxy bulk (Figure 2a–c). At 3 wt % water the water does not fully cover the surface, and some DGEBA are still close to the surface at 3 Å (Figure 2i), although the peak has reduced in height when compared to the dry film (Figure 2b). When water content is increased to 9 wt %, the water forms a complete layer covering the surface and a second (partial) hydration layer can also be seen which displaces the epoxy resin from the surface (Figure 2c). This displacement is consistent with the change in failure mechanism in humid environments from cohesive (within the epoxy resin) to adhesive (at the epoxy‐surface interface). The displacement of the epoxy resin from the surface can also be seen in the radial distribution functions (RDF) (Figure 2d–g) which provide information about the local environment at the surface for the iron in the surface with the three different oxygen atoms, that is, the epoxide (O_epoxy_), the ether (O_ether_), and the water oxygen (O_water_) along with the O_water_−O_water_. At 0 wt % water there is a large peak in the O_epoxy_−Fe RDF at approximately 2 Å and a smaller peak at the same distance in the O_ether_−Fe RDF. These peaks show the close contacts between the Fe and the adsorbed DGEBA. The epoxide group binds more favorably than the ether group due to the difference in charge (O_epoxy_=−0.3343 vs. O_ether_=−0.2962) and the steric hindrance of the aromatic group for O_ether_, in agreement with our previous findings.^[21]^ This can also be seen in Figure 2h–j which shows a snapshot of the oxygen atoms within 4 Å of the surface with O_epoxy_ in blue and the O_ether_ in green (i.e. the closest contacts) where it is clear that more O_epoxy_ are located at the surface. As the water content increases the water displaces the DGEBA from the surface (Figure 2i, j). The O_water_−O_water_ RDF in Figure 2g alongside the snapshots at 3 and 9 wt % water indicate that the water on the surface is fluid‐like and possess no form of long‐range ordering. At 3 wt % the peak at 2 Å in the O_epoxy_−Fe RDF has decreased in height and the peak in the O_ether_−Fe RDF has disappeared whilst a peak at 2 Å appears in the O_water_−Fe RDF. Upon increasing the water to 9 wt %, the peak in the O_epoxy_−Fe RDF at 2 Å also disappears as the water displaces the DGEBA on the surface.

**Figure 3:**
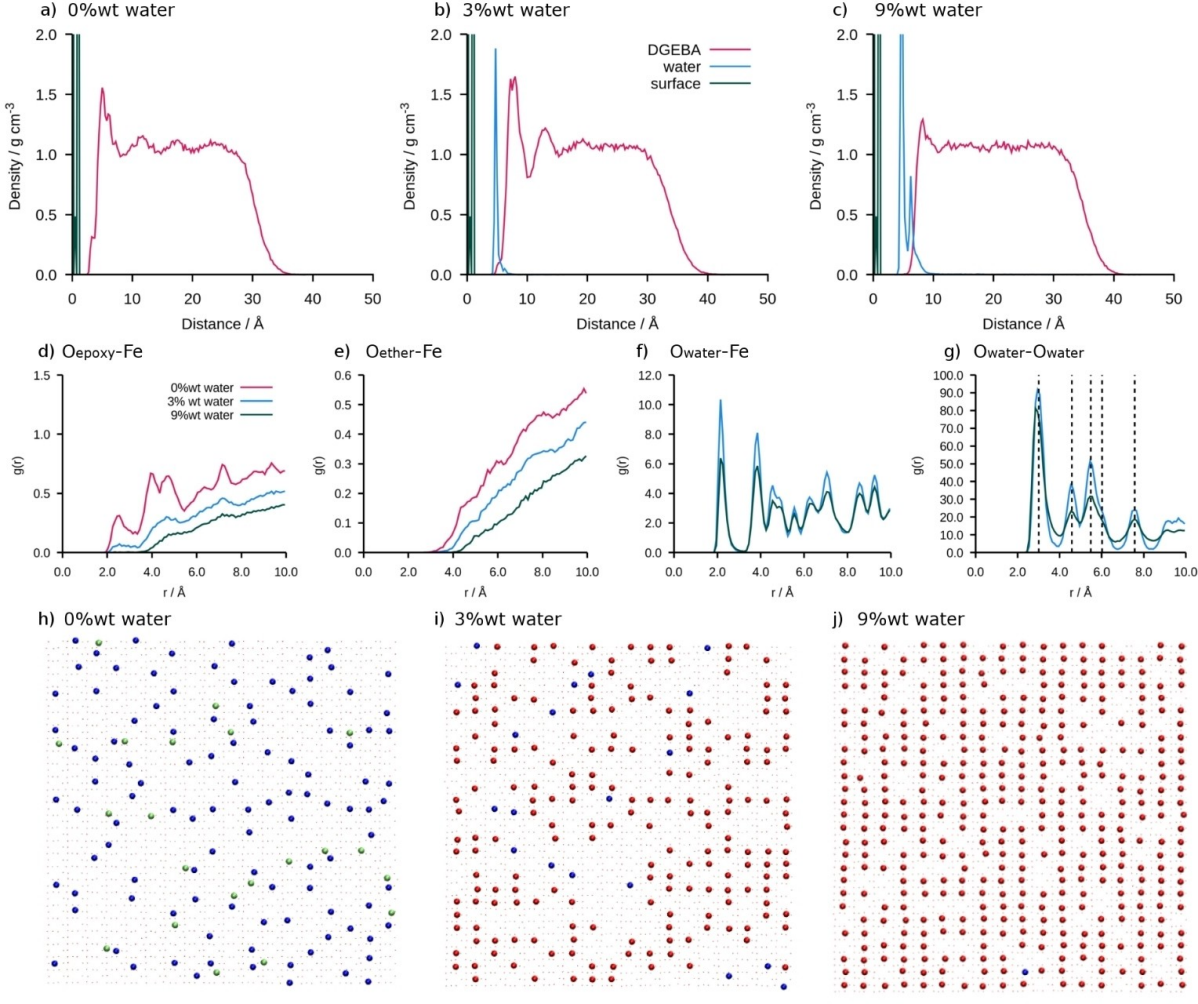
Characterisation of the goethite/DGEBA interface including density profiles, radial distribution functions between selected pairs of atoms, and the snapshots of the position of interfacial oxygen atoms. Density in *z*‐direction for a) 0 wt %, b) 3 wt % and c) 9 wt % water. Radial distribution functions for d) O_epoxy_−Fe e) O_ether_−Fe, f) O_water_−Fe, and g) O_water_−O_water_. Snapshots of the surface oxygen for h) 0 wt %, i) 3 wt %, and j) 9 wt %. The interfacial oxygen are defined as oxygen atoms within 4 Å of the surface. Epoxide oxygen in DGEBA are shown in blue, ether oxygen in DGEBA in green and water oxygen in red.

The results on a goethite surface are qualitatitely different from those on the hematite surface. As in the case of hematite, the water forms a (partial) monolayer between the surface and the DGEBA at 3 wt % water and the formation of a second hydration layer at 9 wt % water. The hydration layers on goethite can be seen in both the RDF (Figure 3d–g) and in the snapshots (Figure 3h–j) which show significant ordering. The O_water_−O_water_ RDF has much sharper peaks than the hematite case. This ordering is due to the structure of the goethite surface where the surface hydroxyl groups enable the electronegative oxygen atoms in DGEBA to sit in a well and form additional hydrogen bonds alongside the electrostatic interactions.^[22]^ We have plotted the distances that correspond to a rectangular lattice with a=4.58 Å, b=3.01 Å in Figure 3g, overlaying the O_water_−O_water_ RDF which correspond to the Fe spacing on the surface, indicating that the water is located in the wells above the Fe sites.

In addition to the formation of a structured water layer in goethite, we can identify some important differences in the film properties of the two materials: (1) we see water displacing the O_ether_ atoms in goethite similarly to what is seen in hematite, but we don‘t see any peak in the O_ether_−Fe RDF for the goethite dry film as seen in hematite, and (2) we see that the initial peak in the DGEBA density has increased in height from 1.55 to 1.65 g cm^−3^ at 3 wt % water on the goethite surface indicating that the epoxy precursor has become compressed at the surface. This is contrary to the behaviour observed for hematite which sees the first peak decrease with any water present. By 9 wt % water the first peak in the DGEBA density on both surfaces has decreased and been displaced from the surface by the water.

The oscillations in the density of the epoxy near the solid interface are characteristic of the formation of an interphase, that is, a region of the polymer with different properties from the bulk.[^22^, ^31^, ^32^] It has previously been suggested that this interphase is responsible for increased residual stresses causing adhesion failure,^[33]^ different compressibility to the bulk changing the mechanical properties,^[34]^ and a deviation of the local glass transition temperature from the bulk.[^34^, ^35^] The range and physical properties of this interphase region are still not well understood and remain an open question, particularly due to the difficulty in experimental measurements of this region. Thin film experiments to mimic the interphase region have found that the interphase can be up to 700 μm in thickness,^[36]^ however advances in infrared microspectroscopy and AFM‐IR have questioned the validity of using thin films as proxies for the buried interface.[^37^, ^38^, ^39^] Conversely, simulation results have found the interphase to be on the order of 1–4 nanometers.^[40]^

Due to the restrictions on simulation size it is not possible to categorically give the thickness of the interphase region for the hematite case, as the fluctuations can still be seen throughout the epoxy for the dry case, whilst they have been dampened by 20 Å for 9 wt % on goethite. These results show that the interphase reaches much further than the influence of the surface on individual molecules, which we found to be around 10 Å in a previous study on single molecules.^[21]^ We postulate that this decrease in interphase region thickness is down to the hydration layers shielding the epoxy precursor from the surface. We investigated the ordering by considering the central quaternary carbon atom and found ordering only in the *z* direction perpendicular to the interface. This ordering mirror that of the bulk density along *z* (Figure 2 and Figure 3a–c and Supporting Information).

**Figure 4:**
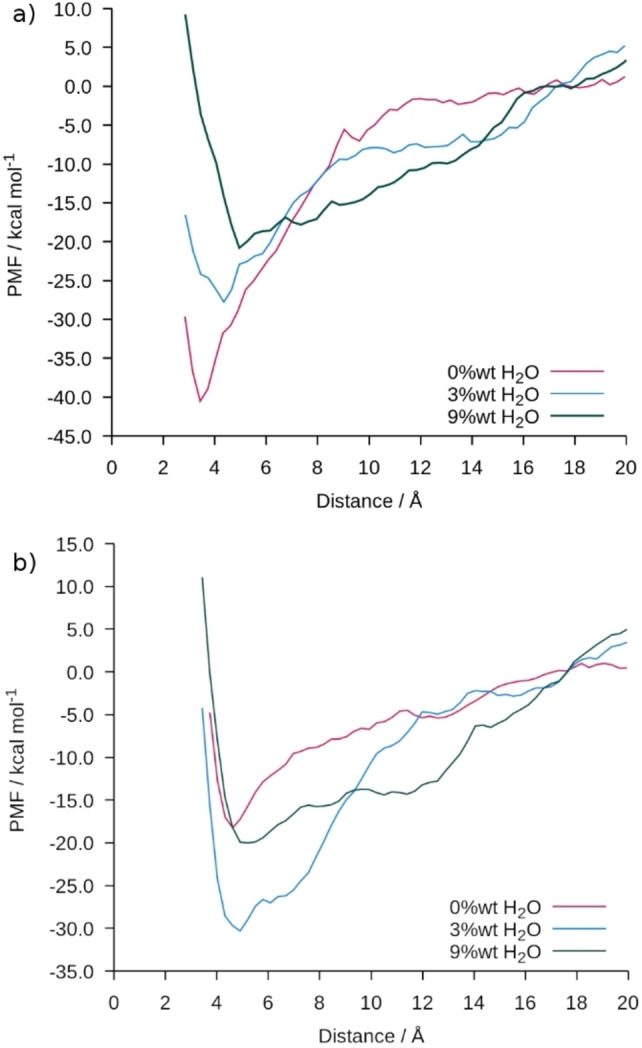
PMF for DGEBA being removed from the surface through a DGEBA film on a) hematite and b) goethite wilth 0, 3, and 9 wt % water at 300 K.

We then calculate the potential of mean force (PMF) to remove a single DGEBA molecule from the surface at 300 K shown in Figure 4. In the case of hematite, we find that the PMF well depth decreases with increasing moisture content, suggesting that the epoxy molecules bind less tightly to the surface in the presence of water. The location of the minima also changes from 3.43 to 4.95 Å as water displaces the epoxy molecules, in agreement with the unconstrained simulations at 350 K. For goethite, however, the addition of a small amount of water (3 wt %) increases the adhesion. This is due to the contraction and increased density in the DGEBA film found in Figure 3b. The width in the PMF at 3 wt % water is significantly larger than for the dry film, approximately 10 Å which corresponds to the first peak in the DGEBA density distribution, whilst the position of the well minimum is the same as in the dry case. At 9 wt % water a second hydration layer has formed for both hematite and goethite surfaces which shields the DGEBA from the influence of the solid surface and the PMFs in both cases are very similar.

## Conclusion

The analysis of the structure of the interface and the interphase region, as combined with the PMF calculations show a qualitatively different behavior on hematite and goethite surfaces as models for a steel or iron substrate. On both surfaces the water lies at the interface between the epoxy precursor and the solid. On the hematite the water displaces the DGEBA from the surface, decreasing the interaction and reducing the free energy of adhesion. However, for goethite the presence of a small amount of water increases the free energy of adhesion as the water causes the density of the DGEBA to increase at the surface. By 9 wt % water this enhancement has been overcome by a second hydration layer and both surfaces have a very similar potential of mean force. These results provide insight into the role of water at the interphase region and highlight the complex nature of epoxy adhesion on steel substrates. Water ingress drastically changes the adhesion properties at the surface for an epoxy precursor and increasing our understanding of these systems at the molecular level can aid in the formulation of coatings and adhesives.

These results also further explain why the development of suitable tests which results in reliable prediction of coating corrosion protection performance has proved to be very time consuming. It is well understood that in the case of steel the “rust” formed on the steel surface is in fact a complex mixture of different oxide/hydroxides/oxy‐chlorides dependent on the environment and as such specific tests have been developed for specific conditions. This work further reinforces not just the importance of developing the correct rust, but also the role which the environment plays at the coating–steel interphase.

## Conflict of interest

The authors declare no conflict of interest.

1

## Supporting information

As a service to our authors and readers, this journal provides supporting information supplied by the authors. Such materials are peer reviewed and may be re‐organized for online delivery, but are not copy‐edited or typeset. Technical support issues arising from supporting information (other than missing files) should be addressed to the authors.

Supporting Information

## Data Availability

The data that support the findings of this study are available from the corresponding author upon reasonable request.
