# Supplementary material for: Water‐Mediated Epoxy/Surface Adhesion: Understanding the Interphase Region
Source: Chemistry. 2022 Oct 1;28(68):e202202483. doi: 10.1002/chem.202202483 (PMC10091763; doi:10.1002/chem.202202483)
Supplement: Supplementary file 1 — Supporting Information [file CHEM-28-0-s001.pdf]

Chemistry–A European Journal

Supporting Information

Water-Mediated Epoxy/Surface Adhesion: Understanding the Interphase Region

Charlie R. Wand,* Simon Gibbon, Peter Visser, and Flor R. Siperstein*

Computational Methods

We performed molecular dynamics simulations of DGEBA and water on hematite (0001) and goethite (100) surfaces using LAMMPS[1]. The two surfaces were picked as they are the most thermodynamically stable [2,3]. Note that in the case of goethite, we are using the *Pnma* space group. Other papers have used the *Pbnm* space group. The face we use corresponds to the (010) surface in the *Pbnm* group[4]. The CLAYFF force field was used to model the solid surfaces[5], with the modification by Kerisit to account for octahedrally coordinated iron[6]. The DGEBA were modelled using the OPLS-AA force field[7] with the C-H bonds constrained using the SHAKE[8] algorithm. The charges on the DGEBA were calculated using LigParGen[9] and the 1.14* CM1A utility[10] and averaged over equivalent sites. Water is represented using the SPC water[11]. In all cases standard Lorentz-Berthelot mixing rules are used to represent cross interactions with a cut off of 10Å. Long range electrostatic interactions were handled with a standard particle-particle-particle-mesh solver[12].

The solid surfaces were prepared by replicating crystallographic information along three Cartesian coordinates. This resulted in a box of x=82.165Å, y=71.1567Å for hematite and x=82.440Å, y=81.346Å for goethite. In both cases a large z direction (200Å) was used to ensure that periodic images did not interact. The hematite slab is 27Å thick, whilst the goethite slab is 26Å thick. The starting configurations were prepared using PACKMOL[13] and MOLTEMPLATE[14]. In all cases the water was randomly inserted between 0-5Å of the surface. We investigated 0, 3 and 9%wt water which corresponds to 0, 204 and 612 water molecules respectively. 360 DGEBA molecules are then inserted at random between 5-55Å.

Preliminary tests with a fixed surface showed that by inserting the water molecules between the DGEBA and the surface fewer water molecules escaped the system around the periodic boundary in the z-direction to adsorb on the underside of the slab (i.e. exposed without an epoxy coating). In all cases more than 96% of water molecules remained on the uppermost surface either between the DGEBA and slab or within the DGEBA coating itself. However, if the simulations are repeated starting from a randomised mixture of water and DGEBA on the surface, a large amount of the water evaporated around the periodic boundary conditions and adsorbed to the underside (bare solid surface) of the slab. Therefore, the amount of water in the solid slab and DGEBA system was unable to be accurately controlled and this case and was significantly less than expected for the case of starting from a random mixture.”.

All systems were run for 25000 steps increasing the temperature from 0.1K to 350K before a longer equilibration run at 350K for $5x10^6$ steps. By running at a slightly higher temperature it allowed the system to fully equilibrate, however, the temperature was not so high that the water evaporated and remained on the topside of the slab.

Umbrella sampling was used to calculate the potential of mean force (PMF) and implemented using PLUMED[15]. A harmonic potential with the form;

$$U(\lambda) = \frac{k_{bias}}{2}(\lambda - \lambda_0)^2 \tag{1}$$

Where λ is the reaction coordinate, here taken to be the distance of the centre of mass of the probe molecule from the top of the surface, k_{bias} is the biasing constant and λ_0 is the value at which $U(\lambda)$ = 0.For these calculations, we take k_{bias} = 21 $kcal\ mol^{-1}$. We calculate the PMF from 2.5Å to 20Å using a

window spacing of 0.3Å. The unbiased PMF is then reconstructed using the weighted histogram analysis method (WHAM)[16]. Each window is run for 5ns, with the last 2ns of the run used for the analysis.

To prepare the starting configuration for the umbrella sampling we perform a series of steered molecular dynamics simulations. These utilised the harmonic potential given in Equation 1 with k_{bias} = 100 $kcal\ mol^{-1}$ and gradually varied λ_0 at a speed of 33 Å/ns. To avoid overlaps in the initial configurations, the probe molecule is initially pulled from 40Å above the surface to 0Å at the surface before the starting configurations are created pulling the probe away from the surface. Both the steered MD and umbrella sampling is done at 300K. In these simulations the bottom 30% of the slab is held fixed (i.e. not integrated through time) to stop the entire system drifting and moving in the z direction with the harmonic restraint.

Model parameters

Table 1 Parameters employed in the molecular dynamics simulations. Taken from the OPLS-AA force field, CLAYFF force field and Kerisit[5–7]. The water model is SPC.[11] Standard Lorentz-Berthelot mixing rules were used for cross-interactions.

Atom type	ε / kcal mol $^{-1}$	σ / Å	Charge
**Alkyl C**	0.066	3.50	-
**Alkyl H**	0.030	2.50	-
**Aromatic C**	0.070	3.55	-
**Aromatic H**	0.030	2.42	-
**Ether/Epoxy O**	0.140	2.90	-
**O in water**	0.1553	3.166	-0.820
**H in water**	0.00	0.00	0.410
**Fe**	9.0263×10^{-6}	4.072218	1.575
**O in iron oxide**	0.1554	3.165541	-1.050
**O in OH in goethite**	0.1554	3.165541	-0.925
**H in OH in goethite**	0.000	0.00	0.425

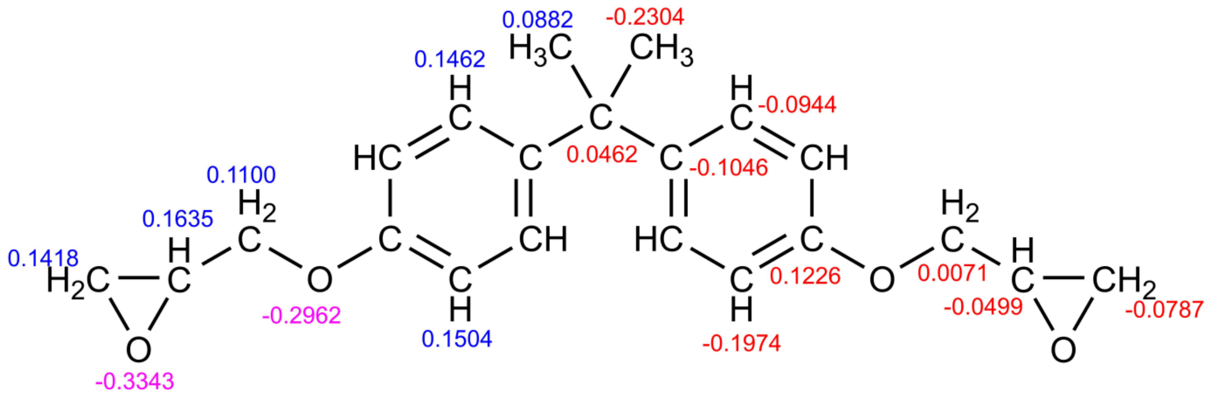

Figure 1 Sketch of DGEBA showing the charges assigned for each atom environment. For ease of clarity C charges are given in red, H in blue and oxygen in pink. The charges are assigned using 1.14*CM1A.

Model validation

Preliminary analysis of the systems at 300K show that the density of the thin epoxy layer is 1.11 g cm^{-3} in good agreement with the experimental density at room temperature (1.16 g cm^{-3} [17]). The density at 350K is slightly lower (1.07 g cm^{-3}) and consistent for all systems. In all cases the epoxy-air interface is characterized by a slope, giving a film thickness of around 30-35Å. At 300K all the water in the 3%wt case is located at the interface for both surfaces, however at 9% there is approximately 3-4 water molecules in hematite, or 0.054%wt, and 5-6 water molecules on goethite (0.06%wt%wt) which is in excellent agreement with experimental data[18] which has a maximum moisture content of 0.07%wt.

Comparison between 350K unconstrained simulations and 300K Umbrella Sampling (US) simulations

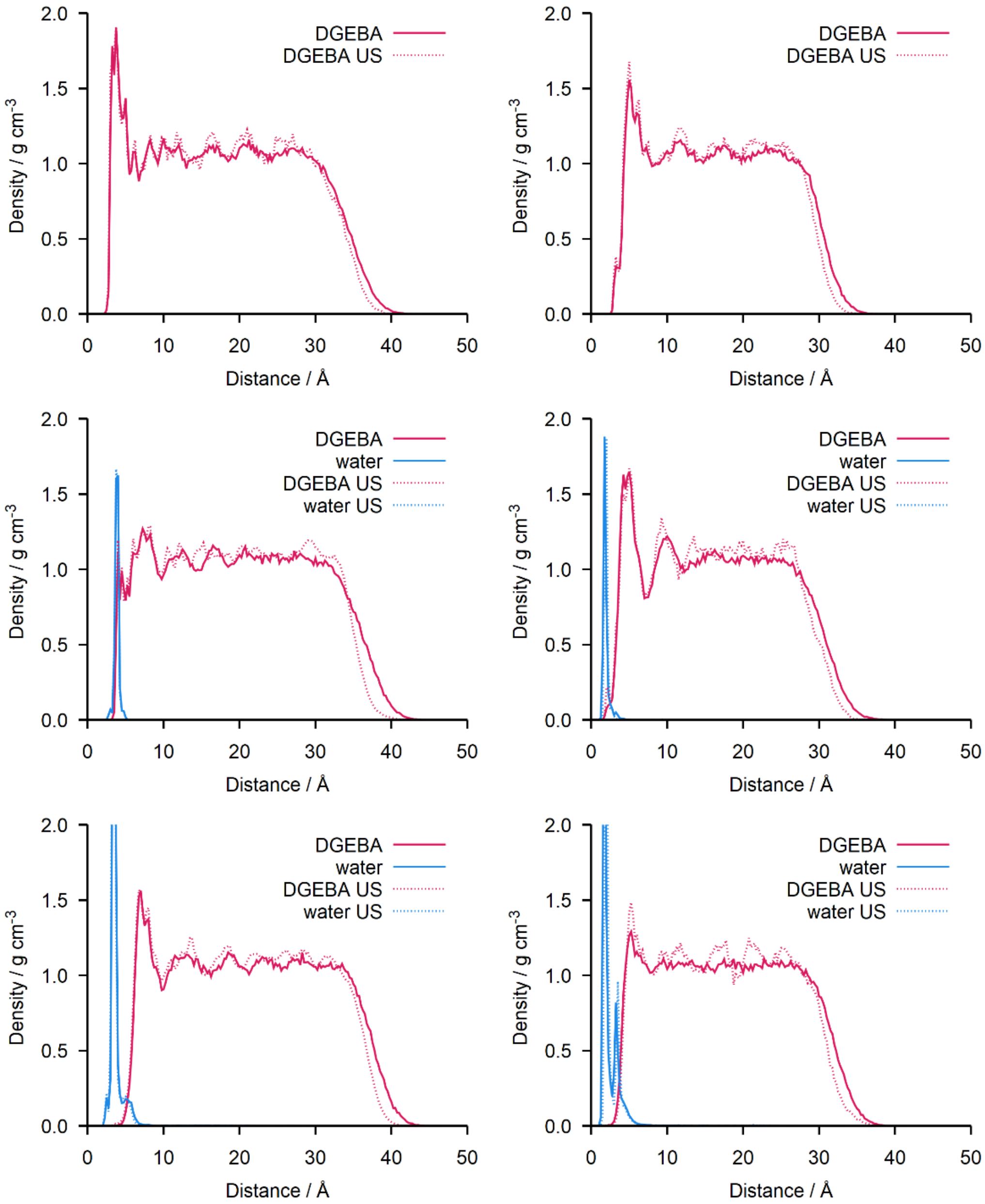

Figure 2 Concentration density of DGEBA and water in z-direction comparing results from the 350K unconstrainted simulations (solid line) and 300K umbrella sampling simulations for the window at the PMF minima. Left: hematite, Right: goethite. Top: 0%wt, middle 3%wt and bottom 9%wt water.

Ordering parallel to interface

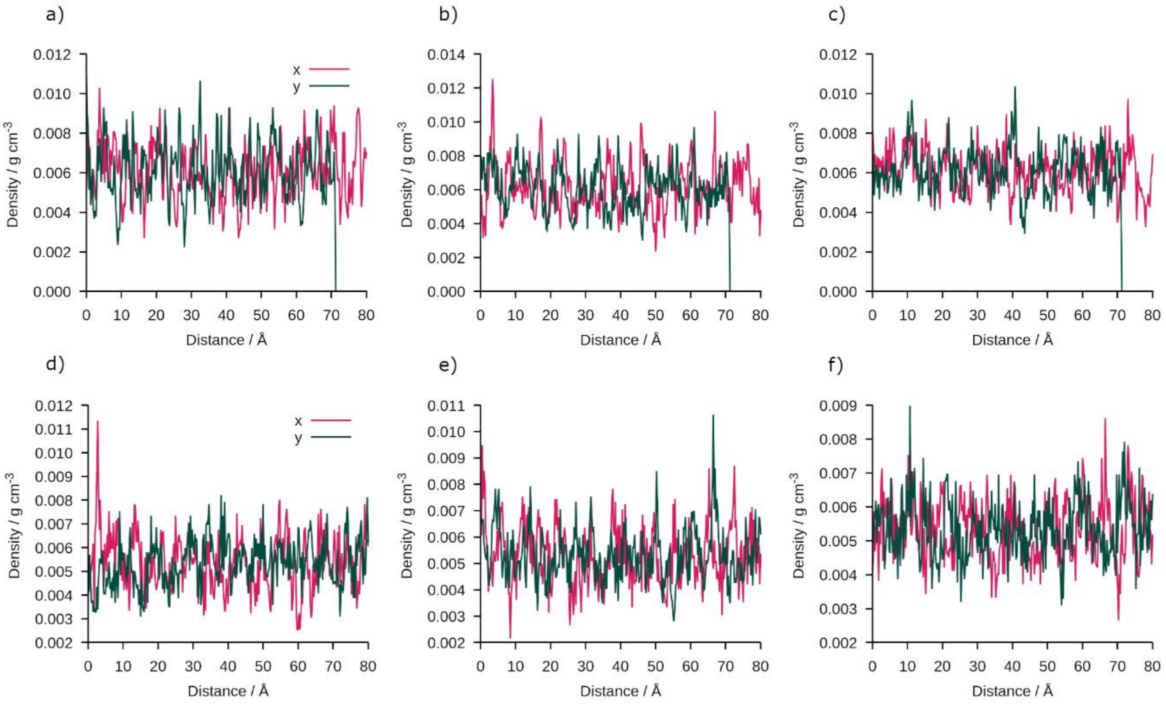

Figure 3 Concentration density of the quaternary central carbon in DGEBA along x and y, parallel to the surface for hematite (a-c) and goethite (d-f) for 0 (a,d), 3 (b,e) and 9 (c,f)%wt water.

References

[1] S. Plimpton, *J. Comput. Phys.* **1995,** *117,* 1-19, DOI 10.1006/jcph.1995.1039

[2] H. Guo, A. S. Barnard, *J. Mater. Chem.* **2011**, *21*, 11566-11577 DOI 10.1039/c1jm10381d.

[3] R. M. Cornell, A. M. Posner, J. P. Quirk, *J. Inorg. Nucl.* **1974**, *36*, 1937-1946 DOI 10.1016/0022-1902(74)80705-0.

[4] S. R. Randall, D. M. Sherman, K. V. Ragnarsdottir, C. R. Collins, *Geochim. Cosmochim. Acta* **1999**, *63*, 2971-2987 DOI 10.1016/S0016-7037(99)00263-X.

[5] R. T. Cygan, J. J. Liang, A. G. Kalinichev, *J. Phys. Chem. B* **2004**, *108*, 1255-1266 DOI 10.1021/jp0363287.

[6] S. Kerisit, *Geochim. Cosmochim. Acta* **2011**, *75*, 2043-2061 DOI 10.1016/j.gca.2011.01.026.

[7] W. L. Jorgensen, J. Tirado-Rives, *Proc. Natl. Acad. Sci. U. S. A.* **2005**, *102*, 6665-6670 DOI 10.1073/pnas.0408037102.

[8] J. P. Ryckaert, G. Ciccotti, H. J. C. Berendsen, *J Comput. Phys.* **1977**, *23*, 327-341 DOI 10.1016/0021-9991(77)90098-5.

[9] L. S. Dodda, I. C. De Vaca, J. Tirado-Rives, W. L. Jorgensen, *Nucleic Acids Res.* **2017**, *45*, W331-W336 DOI 10.1093/nar/gkx312.

[10] L. S. Dodda, J. Z. Vilseck, J. Tirado-Rives, W. L. Jorgensen, *J. Phys. Chem. B* **2017**, *121*, 3864-3870 DOI 10.1021/acs.jpcb.7b00272.

[11] W. J. Meath, D. J. Margoliash, B. L. Jhanwar, A. Koide, G. D. Zeiss, in *Intermolecular Forces Vol 14* (Eds. B. Pullman), Springer, Dordrecht, **1981**, 101-115.

[12] R. W. Hockney, J. W. Eastwood, *Computer Simulation Using Particles*, CRC Press, Boca Raton, **2021**.

[13] L. Martinez, R. Andrade, E. G. Birgin, J. M. Martinez, *J Comput Chem* **2009**, *30*, 2157–2164 DOI 10.1002/jcc.21224.

[14] A. I. Jewett, Z. Zhuang, J.-E. Shea, *Biophys J* **2013**, *104*, 169a DOI 10.1016/j.bpj.2012.11.953.

[15] G. A. Tribello, M. Bonomi, D. Branduardi, C. Camilloni, G. Bussi, *Comput Phys Commun* **2014**, *185*, 604–613 DOI 10.1016/j.cpc.201309.018.

[16] A. Grossfield, "WHAM: the weighted histogram analysis method", http://membrane.urmc.rochester.edu/wordpress/?page_id=126 *version* **2012**, *2*, 6.

[17] J. D. McCoy, W. B. Ancipink, C. M. Clarkson, J. M. Kropka, M. C. Celina, N. H. Giron, L. Hailesilassie, N. Fredj, *Polymer* **2016**, *105*, 243-254 DOI 10.1016/j.polymer.2016.10.028.

[18] Dow Plastics, *D.E.R. 331 Product Information* **2000**, 2.
